# Bereaved Caregivers’ Experiences of End of Life Care For People With Advanced Heart Failure: A Narrative Synthesis

**DOI:** 10.1177/00302228221124636

**Published:** 2022-09-13

**Authors:** Melanie F. J. Diggle, Sue Schutz, Dan Butcher

**Affiliations:** 1 Oxford School of Nursing and Midwifery, Faculty of Health and Life Sciences, 98464 Oxford Brookes University, UK

**Keywords:** heart failure, end of life care, palliative care, caregivers, terminal illness

## Abstract

**Background:**

Heart Failure is a life-limiting condition with a poor and uniquely unpredictable prognosis. The aim of this review is to present and synthesise the current evidence around bereaved caregivers’ experiences of end of life care for people with Heart Failure.

**Methods:**

A systematic review of the literature was conducted using four electronic databases (CINHAL, Medline, BND, PsycINFO). Data was analysed and presented using a narrative synthesis approach.

**Results:**

Eight articles were included within this review. Themes included: Limited and inadequate communication around the condition (including prognosis, preparations for death and the aim of palliative care), the burden of caregiving, and the limited provision of services and formal support.

**Conclusion:**

Bereaved caregivers experience unique and significant challenges when caring for someone dying from Heart Failure. However, further research is required to greater understand the experiences of bereaved caregivers of people with Heart Failure.

## Introduction

Living with Advanced Heart Failure has negative consequences on the quality of life and overall well-being of the person and their informal caregiver (McIlvennan & Allen, 2016). Symptom burden and morbidity resulting from Heart Failure is high, which has been found to place a significant demand on caregivers when enabling a person to live well and die from Heart Failure (Ponikowski et al., 2014). The unpredictable trajectory of Heart Failure renders a unique burden on caregivers. The instability of the pathway of Heart Failure is characterised by periods of acute crisis and decline that may revert to relative stability; unexpected death is not unusual (Hupecy et al., 2009). The burden of ‘seeking normal’ (Penrod et al., 2011), places exceptional demands on the caregiver during end of life care because the Heart Failure death trajectory is not one of gradual decline as may be seen in other long term conditions but one of peaks and troughs (Dionne-Odom et al., 2017).

Palliative Care is a multi-disciplinary approach that aims to enhance the quality of life for all those living with a life-limiting condition (National Council for Palliative Care [NCPC], 2015; World Health Organisation [WHO], 2020). Yet despite this, barriers continue to exist in the accessibility of such services for people with Heart Failure (Hupcey et al., 2009; Sobanski et al., 2020), despite clear recommendations that palliative care is offered to people with Heart Failure on a par with cancer and other long term conditions (Crespo-Leiro et al., 2018). Instead, the burden and responsibility of providing care and support for people dying from Heart Failure often falls to that of their informal caregivers (Kitko et al., 2020), who often have little understanding of what they or their loved one should expect from health care services at the end of life (Hupcey et al., 2016; Alonso et al., 2017; Dionne-Odom et al., 2017).

## Background

### What is Heart Failure?

Heart Failure is a complex syndrome that occurs due to anatomical and physiological abnormalities of the heart (National Institute of Health and Care Excellence [NICE], 2018). It is a life-limiting condition with a debilitating symptom burden that usually includes dyspnoea, fatigue, oedema and pain (McMurray et al., 2013). Heart Failure is often described as the final common pathway for a variety of different cardiac conditions that affect the ventricular function of the heart (Department of Health [DoH], 2000). As management processes have improved for these conditions, so has the prevalence of people living with Heart Failure (National Health Service Improving Quality, 2017). In the United Kingdom (UK), 920,000 people are thought to be living with Heart Failure, with 200,000 new diagnoses every year (Conrad et al., 2018). Despite developments in therapeutic interventions, Heart Failure continues to be a progressive and fatal condition for which there remains no cure (National Health Service Improving Quality, 2017). The 5-year survival rate for people with Heart Failure is 48%, which indicates a shorter life expectancy than many other long-term conditions and malignant diseases (Taylor et al., 2019).

### Palliative and End of Life Care

Development of the speciality of Palliative Care within the UK was pioneered by Cicely Saunders in the 1960s, her contribution during this time was to focus on the terminal stages of malignant diseases when all attempts at curative interventions had concluded. As such she became key in defining a new knowledge base regarding Palliative Care, working tirelessly to relieve the suffering of those dying from Cancer (Clarke, 2007). Palliative Care today can be considered as a broadly inclusive term that aims to enhance the quality of life of people living with any life limiting condition (World Health Organisation [WHO], 2020). Palliative care has been increasingly identified as a key recommendation within the management of all chronic long-term conditions, with recent policy and guideline development within Heart Failure supporting this (Department of Health [DoH], 2008; NICE, 2018; National Partnership for Palliative and End of Life Care, 2015; National Health Service Improving Quality, 2017).

### Palliative and End of Life Care for People with Heart Failure

Evidence suggests that Palliative Care when combined with Heart Failure specialities can make a positive contribution to the way in which the condition is managed (Ponikowski et al., 2014). Research has found that such integration between specialities can facilitate positive outcomes for those living with Advanced Heart Failure (Diop et al., 2017; Talabani et al., 2020). Recently, there have been increased efforts to improve and encourage collaboration between teams to sufficiently integrate care (Kavalieratos et al., 2017; Lewin & Schaefer, 2017). However, evidence indicates that only 4–7% of people living with Heart Failure receive Palliative Care input (Gadoud et al., 2014). Indeed, caregivers caring for a person with the less well understood Heart Failure with Preserved Ejection Fraction (HFpEF) are particularly at risk of missing out on palliative and end of life care because they do not have access to specialist medical and nursing management and rely on general practitioners to coordinate their care who, are likely to lack highly specialised knowledge (The Heart Failure Policy Network, 2020). Such limited involvement from palliative and end of life care would indicate that people with Heart Failure may be spending their final days heavily reliant on the support of their informal caregivers without specialist input. Therefore, the research question of this review is: What are bereaved caregivers’ experiences of end of life care for people with Advanced Heart Failure?

## Methods

### Aim

This narrative synthesis aims to present and synthesise the current evidence around bereaved caregivers’ experiences of end of life care for people with Heart Failure.

### Design and Methods

A systematic review of the literature using a narrative synthesis approach was undertaken. Narrative synthesis is a recognised approach to the systematic review and synthesis of findings from multiple studies to present and discuss within a narrative format (Popay et al., 2006; Ryan, 2013). A narrative synthesis was selected due to the breadth of the studies included within this review that made them insufficiently similar for meta-analysis. Additionally, this approach was selected due to the complexity of the factors involved in bereaved caregivers’ experiences of end of life care, which also permitted for the inclusion of a wider variety of existing evidence to answer the research question and form a broader understanding (Popay et al., 2006; Ryan, 2013).

### Search Strategy

Electronic searches were undertaken between January 2021 until July 2021, the search was updated in March 2022. Databases electronically searched using keywords and associated subject headings included: Medline, British Nursing Database, Cumulative Index to Nursing and Allied Health Literature (CINAHL) and PsychINFO. These databases were selected due to their breath of multi-disciplinary publications that may assist in answering the research question. Furthermore, these databases search across both national and international peer-reviewed journals that may provide relevant empirical research. In addition to database searches, citation tracking was conducted via the use of Web of Science and hand-searching the reference lists of the included articles. Grey literature, namely conference abstracts were not included in this review as they could not be appraised for methodological quality.

### Keywords

The following keywords and associated subject headings are presented in Table 1.

**Table 1.** Keywords and Associated Subject Headings.

Keyword concept separated by ‘AND’	Bereaved	Caregiver	End of Life	Heart Failure
Keyword synonyms separated by ‘OR’	Bereave*, loss, grief, grieving	Carer*, caregiver*, family, families, spouse, partner, friend, “next of kin”, NOK, “supportive person”, supporter*, “loved one”	“end of life care”, “palliative care”, “terminal care”, “terminally ill”, EoLC, death, dying	“heart failure”, “cardiac failure”, “congestive heart failure”, “chronic heart failure” “advanced heart failure” HF, CF, CCF, CHF, HFrEF, HFmrEF, HFpEF

### Inclusion and Exclusion Criteria

The inclusion criteria for this review were:

- Papers published in the English language
- Peer reviewed primary research
- Papers published post the year 2000
- Papers which focused on bereaved caregivers (informal and over the age of 18 years) and their experiences of end of life care for people with Heart Failure.

Studies were excluded if they did not meet all of the inclusion criteria outlined above and/or focused on end of life care for those with left ventricular assistance devices due to specific condition complexities, such as decisions involving device deactivation.

### Search Outcome

**Figure 1.**
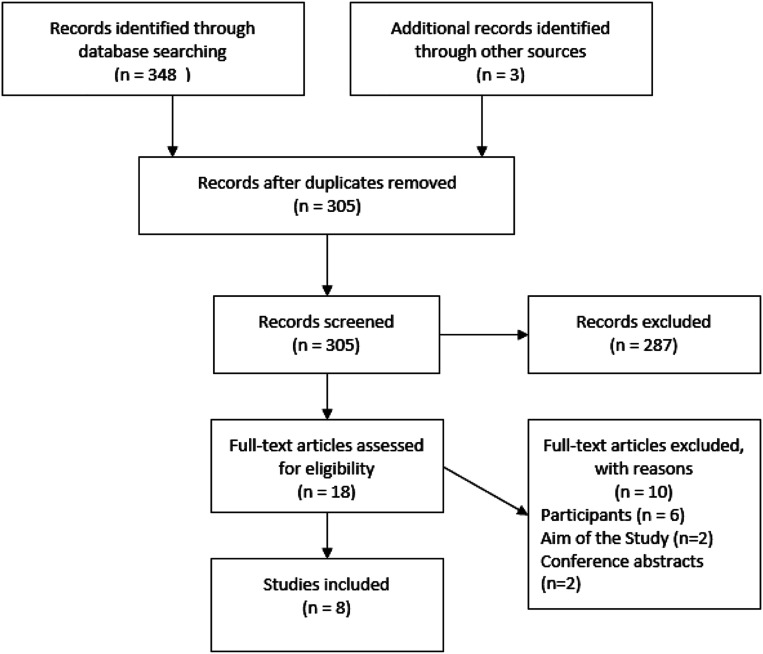
Search strategy.

Three hundred and five articles were screened, of which eighteen articles were eligible for full-text review. Eight articles met the inclusion and exclusion criteria and were subsequently included for analysis within this review. Figure 1 displays the PRISMA flow diagram which represents the search strategy, this has been adapted from Page et al. (2021).

### Methodological Quality

All the papers included within this review were critically appraised and assessed for methodological quality using either the Critical Appraisal Skills Program (CASP) checklist for qualitative research (CASP, 2018) for those studies that were qualitative in nature or the Mixed Methods Appraisal Tool (MMAT) (Hong et al., 2018) for those studies that employed a mixed methods approach. No studies were excluded following critical appraisal. All of the studies met the minimum appraisal criteria with the majority demonstrating the presence of quality indicators as outlined in the relevant assessment domains. Where there were potential study limitations from a methodological quality perspective, these have been considered within the narrative synthesis and subsequent discussion of this paper (Booth et al., 2012).

### Data Extraction and Synthesis

Data was extracted, analysed and synthesised following the guidance by Popay et al., 2006. Popay et al., 2006 advised that when developing a preliminary synthesis of qualitative findings of the included papers an approach that seeks to ‘translate’ the data should be considered. Following the repeated reading of each of the included papers, themes for each paper were identified and recorded in Table 3. Themes from each of the papers were grouped under ‘Key Themes’ and ‘Sub-Themes’. These themes were then cross-checked and agreed on by the authors. The similarities and differences were then systematically compared for patterns (Ryan, 2013). This narrative synthesis is therefore, organised by comparisons between the included individual study findings, which are presented in the format of answers to the review question as recommended by Ryan and Hill (2016).

## Results

Of the eight studies included, only five papers focused specifically on the experiences of bereaved caregivers of people with Heart Failure. Two additional studies included a mix of long-term conditions which included Heart Failure as a minority of the sample. One study looked at Heart Disease which is a term used to refer to different conditions that affect the structure and the function of the heart muscle, such as Heart Failure (British Heart Foundation [BHF], 2022). Studies were predominantly undertaken within the UK, Northern Ireland and Republic of Ireland (*N* = 5). Of the included studies, five used a sample of only bereaved caregivers, the remainder used a sample that included both active caregivers and caregivers who had been bereaved. Within these papers, only the data that was collected from the bereaved caregiver sample was extracted for this review. The sample sizes of bereaved caregivers, within the included papers ranged from 4 to 105. Across the samples, bereaved caregivers were mostly spouses or children of the person with Heart Failure and predominantly identified as female. Common key themes across the included studies were: limited and inadequate communication, burden of care, and limited provision of services and formal support. Tables 2 and 3 outline the characteristics of each included paper within this review and the identified key themes and sub-themes within each paper.

**Table 2.** The Characteristics of Each Included Paper.

Article no.	Author/Year/Country	Study Aim	Sample	Methods	Findings
1	Boyd et al. (2004) United Kingdom	To describe how people with HF and their caregivers’ view and experience health and social care in the last year of life	112 participants from the UK specifically Southeast Scotland	Qualitative: A prospective, longitudinal study using semi-structured interviews and focus groups at 3 monthly intervals. Only interviews were undertaken with the bereaved caregivers’ sample, 8–12 weeks post death of the person with HF.	Caregivers felt unsupported by services and had limited understanding of heart failure as a condition, with poor communication around treatment aims or prognosis. Psychosocial care for both the person and the caregiver was considered generally poor as was education around the condition and co-ordination of care. Many patients had no access to heart failure nurses and palliative care was rarely available
			Patients *n* = 50		
			Active informal carers *n* = 27		
			Bereaved informal carers *n* = 5		
			Professionals *n* = 30	Data analysed using a narrative analysis framework	
2	Fried and O’Leary (2008) United States of America	To understand how the EoLC experiences of older patients and their caregivers can inform the development of new approaches to ACP	64 bereaved caregivers of people over the age of 60 who had died of advanced cancer, COPD or HF.	Qualitative: Semi-structured interviews with bereaved caregivers 6 months post-death. Analysis was conducted using the constant comparative method	Themes included: The lack of availability of treatment options which impacted broader EoLC issues, changes in preferences at the very end of an illness, variability in patient and caregiver desire and readiness to hear information, difficulties with patient and caregiver communication
			Cancer *n* = 52		
			COPD *n* = 27		
			Heart failure *n* = 21		
3	Small et al. (2009) United Kingdom	To add to the knowledge concerning EoLC for people with HF by assessing carers’ views on EoLC, the circumstances of death and bereavement experiences	20 bereaved caregivers of people from HF from the UK recruited from a larger quantitative longitudinal study	Qualitative: In-depth semi-structured individual interviews with 20 bereaved caregivers of people with HF	Findings were grouped into three time periods: Prior to death, the death itself and bereavement. Communication about death and prior to it occurring was difficult. Dissatisfaction around patients receiving what caregivers considered to be futile treatments. Caregivers adopted a range of coping strategies to deal with grief but had limited formal support
			Female *n* = 17		
			Male *n* = 3		
			Spouse of the person with HF *n* = 13		
			Child of the person with HF *n* = 7		
			Under the age of 60 *n* = 7		
			Between the ages of 60–70 *n* = 5		
			Over the age of 80 *n* = 8		
4	McIlfatrick et al. (2018) United Kingdom and Republic of Ireland	To identify psychosocial factors associated with caregiver burden and evaluate the support needs of caregivers in advanced heart failure	30 participants overall	Phase 2 (qualitative methodology) of a mixed-methods study. Semi-structured one-to-one interviews with current and bereaved caregivers for people with HF. An inductive analysis of the data was undertaken using thematic analysis	One overarching theme and four sub-themes relating to caregiver’s support needs were identified. The overarching theme was the need to plan for the future. The four sub-themes included: Seeking emotional support from someone who understands, information on prognostication, knowledge on how to and where to get support and knowledge on what to expect at the end of life
			Current caregivers *n* = 20		
			Bereaved caregivers *n* = 10		
			Majority of bereaved caregivers had cared for a parent with HF (*n* = 8)		
5	Soroka et al. (2018) United States of America	To elicit the views, feelings and experiences of primary caregivers who provide unpaid care to dying family members in the home setting	16 bereaved caregivers	Qualitative: Exploratory, cross-sectional design using semi-structured interviews. Data analysis was based on the narrative research pedagogy	Four storylines were identified, these included: Values/relationships, stories of terminal illness, needs and support. Caregivers’ confidence is shaped by the terminal illness of the person they care for, as well as the caregivers’ values and relationships. It is also influenced by their needs and the sources of support they receive
			Female *n* =11		
			Deceased person died of cancer *n* = 8		
			COPD *n* = 2		
			Dementia *n* = 2		
			HF *n* = 3		
			Spouses of deceased person *n* = 10		
			Child of deceased person *n* = 6		
			Age range: 20–90 years		
6	Fitzsimons et al. (2019) United Kingdom and Republic of Ireland	To explore caregivers’ experience when caring for a loved one with advanced HF at the end of life and to identify any unmet psychosocial needs	30 participants overall	Qualitative aspect of a larger mixed methods study. Semi-structured one-to-one interviews with current and bereaved caregivers of people with HF. Data analysed using thematic analysis and an inductive approach	Three main themes identified: Poor communication, living with uncertainty, lack of service provision. These themes were supported by six sub-themes: Inadequate understanding of palliative care, a 24/7 physical burden, emotional burden, inability to plan, no care continuity, dying lonely and unsupported
			Current caregivers *n* = 20		
			Bereaved caregivers *n* = 10		
			Bereaved caregivers from the UK *n* = 10		
			Bereaved caregivers female *n* = 10		
			Bereaved spouse of person with HF *n* = 1		
			Bereaved child of the person with HF *n* = 8		
			Bereaved niece of the person with HF *n* = 1		
7	Chester et al. (2021) United Kingdom	To understand and explore the experiences of patients and bereaved carers of patients that were enrolled into the pilot intervention of a combined palliative care and specialist heart failure nurse led service	8 participants overalls	Qualitative: Focus groups, one group of HF patients and two groups of bereaved caregivers of people with HF. Data analysed using thematic analysis	Responses were mapped into four key areas: The experience of being diagnosed with HF and living with the condition, perceptions of referral to PC, key components of the service they found helpful and unhelpful. Within these four areas themes emerged including: Multimorbidity and understanding of HF and its trajectory, communication around referral to PC, having a ‘broker’ for the system, recognition of carer’s needs, responsiveness of the service, and feeling ‘in control’
			Patients living with HF *n* = 4		
			Bereaved caregivers *n*= 4		
			All bereaved caregivers were female		
			Bereaved spouse of person with HF *n* = 3		
			Daughter of the person who had died of HF *n* = 1		
8	Robinson et al. (2021) New Zealand	To explore the impact of uncertainty on how death is remembered by bereaved family members of people with heart disease	A sample of 105 participants who completed the postal survey indicated they had cared for someone at the end of their life with heart disease	Thematic analysis of free text collected during a postal survey of bereaved family’s experiences of healthcare services in the last three months of their life using the New Zealand version of the VOICES questionnaire was undertaken	Analysis of the date identified that when uncertainty regarding the prognosis and management of heart disease is not communicated clearly, physical deterioration and death can appear unexpected to the family. This had a significant impact on the bereaved family. Four key themes related to the impact were identified which included: Distrust in healthcare professionals, stories left incomplete, loss, regret and missed opportunity and, disempowerment

*Note*. EoLC = End of Life Care; HF = Heart Failure; ACP = Advanced Care Planning; HNS = Hospice Nurse Specialist; PC = Palliative Care; IPA = Interpretative Phenomenological Analysis; VOICES = Views of Informal Carers Experiences Survey.

**Table 3.** The Key Themes and Sub-Themes Identified Within the Included Papers.

Key Themes	Limited and Inadequate communication			Burden of Care		Limited provision of services and formal support					
Sub-Themes	Limited understanding of the aim of Palliative Care	Poor understanding of Heart Failure	Preparing for death	Physical, emotional, psychosocial, financial burden	Caregiving as a ‘24/7’ role	Lack of service provision	Inadequate care continuity	Incompatibility of services with end of life care needs	Feeling supported	Dying lonely	Limited formal bereavement support
Boyd et al., 2004		*	*	*		*	*		*		
Fried & O’Leary, 2008			*								
Small et al., 2009		*	*					*			*
McIlfatrick et al., 2018	*	*	*			*			*		
Soroka et al., 2018			*						*		
Fitzsimons et al., 2019	*	*		*	*	*	*		*	*	*
Chester et al., 2021	*	*	*				*		*		
Robinson et al., 2021		*	*	*							

## Themes

### Limited and Inadequate Communication

Limited or perceived inadequacies in communication between caregivers and healthcare professionals, as well as caregivers and the dying person were a common theme. Caregivers across a number of studies reported a limited understanding of the aim and objectives of Palliative Care and how to access it (McIlfatrick et al., 2018; Fitzsimons et al., 2019; Chester et al., 2021). This included a general lack of knowledge about the availability of Palliative Care services for people with Heart Failure (Chester et al., 2021). Caregivers of people with Heart Failure felt that the process of accessing Palliative Care was hindered by their pre-existing misconception that services were only for those who were dying from Cancer (McIlfatrick et al., 2018; Fitzsimons et al., 2019). Furthermore, caregivers reported feeling initially afraid when Palliative Care was suggested as they only associated the service with death and dying (Chester et al., 2021). Caregivers emphasized the importance of healthcare professionals providing information to minimise any uncertainty they were experiencing regarding their family member or friend receiving Palliative Care input (Fitzsimons et al., 2019).

Limited understanding of the aim and objectives of Palliative Care was also compounded by caregivers’ lack of knowledge around the long-term condition of which their family member or friend was dying from. Caregivers of people with Heart Failure often reported lacking a basic understanding of the condition. This included not being able to fully comprehend the nature of the condition and therefore, consequently caregivers had little insight regarding prognosis and the long-term implications of the diagnosis (McIlfatrick et al., 2018; Fitzsimons et al., 2019; Chester et al., 2021; Robinson et al., 2021). Caregivers expressed that the lack of information provided around diagnosis, prognosis, causation and possible management options for Heart Failure was a significant source of uncertainty (Fitzsimons et al., 2019; Robinson et al., 2021). This level of uncertainty contributed to caregivers’ distress, which negatively impacted on the experience of caring for someone with Heart Failure (Fitzsimons et al., 2019). Caregivers identified that their need for information began at the time of diagnosis and continued throughout the trajectory of the disease and into bereavement (McIlfatrick et al., 2018). Furthermore, caregivers’ expressed the view that healthcare professionals should speak openly about the condition and the prognostic consequences to improve their understanding of Heart Failure and support future planning (McIlfatrick et al., 2018; Chester et al., 2021). When communication regarding prognosis was unclear or occurred late into the person’s illness, caregivers reported experiencing loss, regret and identified missed opportunities for important decisions and conversations (Robinson et al., 2021). However, a study by Fried and O’Leary (2008) identified there was significant variability in the amount of prognostic information that caregivers are ready, able and willing to hear. Caregivers within this study spoke of their appreciation for professionals who consulted with them on how much information they wanted. It was therefore, identified that the delivery of such condition specific information should be considered on an individual caregiver basis (Fried & O’Leary, 2008).

The uncertainty caused by a limited understanding of Heart Failure was seen to impact on caregivers’ preparedness for the dying process and the death of their family member or friend (Small et al., 2009; McIlfatrick et al., 2018). Caregivers expressed that in absence of clear explanations about the condition and how death had ultimately occurred, it left many caregivers with unanswered questions (Robinson et al., 2021). Caregivers generally reported feeling unprepared for their family member or friend’s death (McIlfatrick et al., 2018; Robinson et al., 2021). Feeling unprepared for death, contributed to a sense of suddenness, this suddenness in turn gave rise to some caregivers questioning whether or not they had done enough for the person with Heart Failure and in some cases regret that they had not known the person was dying and therefore, had missed the opportunity to be present at the time of their death (Robinson et al., 2021). Overall caregivers expressed wanting knowledge on what to expect as the person neared the end of their life, whilst it was recognised that there were differences between the amount of information each individual caregiver wanted, many sought to know how imminent death would be, in order to be able to plan sufficiently ahead (McIlfatrick et al., 2018). In contrast, some caregivers reported that discussing death whilst their family member or friend was alive was exceedingly difficult and as such was delayed until very late in the illness (Small et al., 2009). Caregivers consistently supported the concept of shared decision making and advance care planning (Fitzsimons et al., 2019; Chester et al., 2021; Robinson et al., 2021) However, caregivers in one study voiced that even with advance care planning there was no explicit consideration given to the caregiver and their wishes within this process (Fried & O’Leary, 2008). Yet despite this, it was felt that healthcare professionals should be supporting caregivers regarding preparations for death and facilitating end of life care discussions (Fried & O’Leary, 2008; Small et al., 2009).

### Burden of Care

Across the studies included with this review, the burden of caregiving as a theme was persistent. Caregivers reflected on their role as being ‘24/7’ with very little relief from their complex responsibilities, highlighting the relentless nature of caring for a dying person (Fitzsimons et al., 2019). Fitzsimons et al. (2019) reported that caregivers’ felt that their difficulties were ‘endless’ and consequently found it difficult to cope. One of the ways in which this manifested itself was through the experience of emotional burden, which was associated with feelings of stress, worry, fear and anxiety (Fitzsimons et al., 2019). Boyd et al. (2004) also noted that low mood and anxiety was prevalent amongst caregivers as they struggled with the daily demands of supporting a person living with Heart Failure. As a consequence, this then negatively impacted on caregiver’s quality of life and overall wellbeing (Fitzsimons et al., 2019). Furthermore, as the person’s condition progressed, caregivers identified an increase in the care and support they needed to provide, of which many felt they had no choice but to take on this extra responsibility even if it was to their detriment (Boyd et al., 2004). In addition, caregivers felt it was their responsibility to recognise when a person’s condition had deteriorated and know when it was appropriate to seek medical attention for this, many caregivers found this challenging with one caregiver describing their experience of seeking assistance for an acutely unwell loved one as ‘frightening’ (McIlfatrick et al., 2018). Robinson et al. (2021) also found that caregivers within their study described feeling an overwhelming sense of responsibility for the person with Heart Failure, and Fried and O’Leary (2008) found that this responsibility included the need to make specific treatment decisions, such as whether to commence or discontinue care which may have otherwise been potentially life-sustaining (Fried & O’Leary, 2008). The studies within this review further reported that caregiver burden was additionally compounded by caregivers’ confidence in their ability to meet the dying person’s needs, with many feeling they did not possess the skills or knowledge to meet the demands of providing care and support to a person nearing the end of their life (Soroka et al., 2018; Fitzsimons et al., 2019).

### Limited Provision of Services and Formal Support

The provision of services and the availability of formal support throughout the end of life phase and subsequent bereavement were a common theme across the studies included within this review. A lack of service provision was reported in the form of delayed referrals, limited timely face to face access with Palliative Care services and a lack of awareness of the availability of such services (Fitzsimons et al., 2019). McIlfatrick et al. (2018) reported that from the bereaved caregivers they interviewed, very few stated that they had received a Palliative Care referral. Furthermore, caregivers reported limited community Palliative Care involvement and when referrals were completed, they were often untimely. For some, Palliative Care was only introduced when the person was nearing the end of their life, with one caregiver reporting that involvement from a Palliative Care team only occurred the day before the person’s death (Boyd et al., 2004).

Across several studies, caregivers described perceived inadequacies in care continuity. This was particularly prevalent in those patients that had multiple co-morbidities and as a result were receiving specialist input from different medical disciplines (Boyd et al., 2004; Fitzsimons et al., 2019; Chester et al., 2021). This was a notable issue for Heart Failure patients whereby, caregivers described the need for better co-ordination between cardiology and specialist Palliative Care teams (Fitzsimons et al., 2019). Where there were multiple disciplines involved, caregivers expressed a lack of understanding and uncertainty about the persons condition and management plan, such as medication regime (Chester et al., 2021). Several caregivers felt that there were too many different and perceived unnecessary interventions from different teams (Small et al., 2009). Furthermore, caregivers commented on the limited consistency regarding which health professionals they saw, with some reporting that they preferred to see the same healthcare professionals (Boyd et al., 2004). A study by Boyd et al. (2004) reported that seeing different doctors caused particular dissatisfaction for caregivers, as they felt that different medical professionals didn’t really know the person living with Heart Failure and were unable to form a professional relationship (Boyd et al., 2004). When relationships between healthcare staff, the person and the caregiver were missing, this was quickly recognised and perceived as a deficiency in staff’s competencies. Resultantly, overall, this affected the caregiver’s confidence in healthcare professionals to adequately care for the dying person and provide them with the necessary support to fulfil their caring role (Soroka et al., 2018).

Where caregivers felt supported by professionals, they described the appreciation of being able to build a partnership that offered them practical and emotional support (Boyd et al., 2004; Small et al., 2009; Chester et al., 2021). Soroka et al. (2018) identified that caregivers who received support in the form of encouragement and positive feedback from healthcare professionals, increased their self-efficacy and confidence in their caring abilities, which was considered to be important in helping them not only fulfil their caring role but also find positive meaning in the experience of caring for a dying family member or friend. Furthermore, caregiver’s confidence in caring for the dying person was identified as being connected with the frequency and quality of the formal support they received (Soroka et al., 2018). This finding is supported by Chester et al. (2021) who identified that as the health of the person with Heart Failure deteriorated, formal support enabled caregivers to ‘feel safe’ and share their caregiving burden with healthcare staff. In contract, caregivers reported frustration when unable to access support even in the simplest format of having a telephone call returned (Chester et al.,2021). Furthermore, many caregivers struggled with knowing how and when to access advice and support during an already difficult time in their lives (McIlfatrick et al., 2018). Caregivers further reported feeling unsupported particularly during the dying phase, with many describing it as a lonely experience, that was both challenging and at times overwhelming due the responsibility of caring for someone in their final days (Fitzsimons et al., 2019). Limited formal support during the dying phase really concerned caregivers with many wishing that someone could tell them what to do. Many caregivers wished that the support in the form of communication from healthcare staff had been better during this phase so that things may have been planned and occurred differently (Fitzsimons et al., 2019)

Whilst it is widely recognised that Palliative Care should not conclude at the point of the person’s death, findings across the included studies suggested otherwise. Two studies included within this review reported that caregivers received little to no bereavement support (Small et al., 2009; Fitzsimons et al., 2019). Some caregivers shared that the death of the person with Heart Failure had left them feeling significantly distressed and that the intensity of their grief had been surprising to them (Small et al., 2009). A number of caregivers sought informal support from friends and family of which they felt was sufficient, yet overall, it was identified that most caregivers would have welcomed the offer of professional bereavement care (Small et al., 2009).

## Discussion

Despite undertaking a comprehensive search of the literature, only eight papers met the inclusion criteria for this review. Within this sample, only five papers either specifically focused on bereaved caregivers of people who had died of Heart Failure others included them as a minority sample within mixed long-term conditions or a Heart Disease demographic. The review findings suggest that bereaved caregivers often have a challenging and poor experience of end of life care for people living with Heart Failure. The reasons for this are multi-factorial. Despite the reported challenges associated with providing care at the end of life, this review has also highlighted that there are positives to be gleaned. Ultimately the negative experiences of caregiving highlighted within this review are inextricably linked with each other. Therefore, to begin to address any one of the issues raised within the presented themes means addressing all of them within the wider context of the provision of end of life care for people with Heart Failure.

Caregivers identified limited and inadequate communication alongside limited understanding of Palliative Care as a service and misconceptions surrounding its aims and objectives. A study by Selman et al. (2007) which included the creation of recommendations for Palliative Care within Heart Failure, found that the caregiver participants when asked about end of life care decisions interpreted the question to be referring to decisions regarding euthanasia or consideration of death by suicide (Selman et al., 2007). A systematic review by Doherty et al. (2016) which considered carers’ needs in Advance Heart Failure, indicated similar findings; recommending that improving awareness of the purpose of Palliative Care services and their availability for long-term conditions such as Heart Failure was required (Doherty et al., 2016). Additionally, within this theme, it was identified that caregivers’ have unmet needs regarding information surrounding Heart Failure as condition, its presentation and prognosis. Doherty et al. (2016) also identified unmet condition specific and prognostic information needs of caregivers of people with Advanced Heart Failure. Research has highlighted the importance of timely and clear communication about the implications of the condition and prognosis, to assist in the management of uncertainty and distress which has been found to exacerbate the burden that many caregivers of people with Heart Failure face (Bekelman et al., 2011; Metzger et al., 2013; Fitzsimons et al., 2019; Walthall et al., 2020). However, across the reviewed literature there was noted variability between caregivers regarding their wish for such detailed information, which is consistent with earlier research (Fried & O’Leary, 2008). Facing the prospect of their family member or friend dying may itself present as a challenge in accepting Palliative Care services and may account for discrepancies between healthcare professionals’ and caregivers’ perceptions of what and how much information was communicated (Kaasalainen et al., 2011). It is, therefore, important for healthcare professionals to consider caregivers’ readiness to receive information regarding introduction of Palliative Care.

The burden of caregiving was a further predominant theme that emerged from the included literature. Providing care for a person dying from Heart Failure affects all areas of caregivers’ lives. The findings from this review are supported by research which has indicated that providing care at the end of a person’s life is associated with significant challenges that can often result in physical, psychosocial, emotional and financial burden (Doherty et al., 2016; McIlvennan & Allen, 2016; Dionne-Odom et al., 2017; McIlfatrick et al., 2018). Furthermore, caregivers within this review often reported having their own healthcare needs which they sometimes were required to neglect because of their caring responsibilities. Difficulties in caregivers maintaining their overall health may lead to a decline in their physical and mental wellbeing, which may in turn incur medical intervention. As well as the clear detrimental impact this has on the caregiver there may also be a negative impact on the person living with Heart Failure, triggering a deterioration in their own health. Combined this could cause an escalation in associated health and social care costs (Doherty et al., 2016; Dionne-Odom et al., 2017). It is, therefore, paramount that caregivers are supported. Within the UK, research to date has identified that caregivers’ support requirements can be classified into two areas, these include: support for caregivers to provide care and support with their own individual well-being (Hardy, 2018). Research has indicated that providing caregivers with support depends on their circumstances and individual requirements (Parker et al., 2016). However, further research is warranted to identify the different ways in which caregivers can be supported in their caring role with consideration as to how such support interventions are then later evaluated, to better understand how caregivers can be optimally supported in caring for a loved one dying from Heart Failure (Doherty et al., 2016; McIlfatrick et al., 2018; Walthall et al., 2020).

Research suggests that the integration of Palliative Care services for people living with Heart Failure can reduce symptom burden and improve quality of life for both the person and their caregiver (Selman et al., 2007). This research has been recognised within the field and as a result many professional and policy organisations have provided recommendations for Palliative Care to be included within Heart Failure management (Jaarsma et al., 2009; McMurray et al., 2013; Yancy et al., 2013; McIlvennan and Allen, 2016). However, the findings of this review indicate that implementation of such services and co-ordination between them remains limited. Research conducted by Doherty et al. (2016) and McIlvennan and Allen (2016) reported similar findings and supported the need for improved collaboration and communication between Palliative Care and Heart Failure specialities. However, this process is complicated by several factors such as: disease uncertainty, multi-morbidity, silos of care and complex treatment decisions (McIlvennan & Allen, 2016). Therefore, further Heart Failure specific research is required to support development within this area of end of life care. Additionally, it is important to consider that the death of the person should not signal the end of Palliative Care involvement for the caregiver, who will continue to have their own support needs throughout their bereavement. Yet, the findings of this review indicate that this is not the case. This has been a similar finding of other studies that have highlighted many bereaved caregivers benefit from professional support to process what has occurred, grieve for their loss and rebuild areas of their life that were neglected due to caring for a dying person (Breen et al., 2014; Hardy, 2018).

### Strengths and Limitations of the Review

Strengths of this paper include the original and contemporary area of exploration and the thorough process that has been followed when undertaking this narrative review. Throughout this paper, the methods used to search, identify and analyse relevant literature have been documented in detail. Limitations of the study include the generalisability of the findings of this review due to a paucity of literature directly related to bereaved caregivers’ experiences of end of life care for people with Heart Failure. Despite a comprehensive search, it only yielded eight papers which met the inclusion criteria for this review and only five of those specifically focused on the condition of Heart Failure. A further limitation of this review is the heterogeneity of the articles included in this study such as the wide range of settings and the location of where the studies were undertaken. In this context it should be noted that differences exist globally regarding healthcare structure and cultural attitudes towards death and dying, which should be taken into consideration when interpreting the findings of this review. The homogeneity of the participants should also be noted, with younger male caregivers being disproportionally under-represented. Furthermore, only articles published in the English language, after the year 2000 have been included therefore, the potential for language and publishing bias also needs to be considered. However, one of the studies was conducted in 2004, in healthcare this is a considerable amount of time ago and as such practices may have advanced since then. When interpreting the findings of this review it is also significant that due to the method of analysis and the utilisation of a narrative synthesis means that the themes generated within this review are based on the data interpretations of other authors.

### Implications for Practice, Research and Education

This review has highlighted that whilst there is a small body of evidence to suggest that care at the end of life for people with Heart Failure and their caregivers needs to be improved, there is overall a lack of empirical research specific to this area. As a result, further research exploring bereaved caregivers’ experiences of end of life care for people with Heart Failure is required. Comprehensive research in this area should aim to inform education for healthcare professionals and support best practice when considering approaches and interventions that support caregivers in their caring role, and overall enhance their experience of supporting a family member or friend who is dying as a result of Heart Failure.

## Conclusion

Undertaking this review has provided a foundational insight into the end of life care experiences of bereaved caregivers of people who have died of Heart Failure. Despite the clear requirement for further research within this field, the papers included within this review have highlighted the need for consideration regarding communication pertaining to the aims and objectives of Palliative Care in conjunction with prognostic and condition specific information. This review has further identified the level of support caregivers may benefit from in the face of the significant burden they experience. In conjunction with the availability and timely access of formal support across the trajectory of Heart Failure; from diagnosis through to and inclusive of bereavement. Therefore, this review has raised awareness about this important area, demonstrating the need for further research to contribute to the evidence base within end of life care for Heart Failure.
